# Reducing Glucokinase Activity to Enhance Insulin Secretion: A Counterintuitive Theory to Preserve Cellular Function and Glucose Homeostasis

**DOI:** 10.3389/fendo.2020.00378

**Published:** 2020-06-09

**Authors:** Nicholas B. Whitticar, Craig S. Nunemaker

**Affiliations:** ^1^Translational Biomedical Sciences Program, Graduate College, Ohio University, Athens, OH, United States; ^2^Diabetes Institute, Heritage College of Osteopathic Medicine, Ohio University, Athens OH, United States; ^3^Department of Biomedical Sciences, Heritage College of Osteopathic Medicine, Ohio University, Athens, OH, United States

**Keywords:** type 2 diabetes, metabolic syndrome, hyperinsulinemia, beta cell, pancreatic islet, insulin, pulsatility, glucokinase

## Abstract

Pancreatic beta-cells are the only cells in the body that can synthesize and secrete insulin. Through the process of glucose-stimulated insulin secretion, beta-cells release insulin into circulation, stimulating GLUT4-dependent glucose uptake into peripheral tissue. Insulin is normally secreted in pulses that promote signaling at the liver. Long before type 2 diabetes is diagnosed, beta-cells become oversensitive to glucose, causing impaired pulsatility and overstimulation in fasting levels of glucose. The resulting hypersecretion of insulin can cause poor insulin signaling and clearance at the liver, leading to hyperinsulinemia and insulin resistance. Continued overactivity can eventually lead to beta-cell exhaustion and failure at which point type 2 diabetes begins. To prevent or reverse the negative effects of overstimulation, beta-cell activity can be reduced. Clinical studies have revealed the potential of beta-cell rest to reverse new cases of diabetes, but treatments lack durable benefits. In this perspective, we propose an intervention that reduces overactive glucokinase activity in the beta-cell. Glucokinase is known as the glucose sensor of the beta-cell due to its high control over insulin secretion. Therefore, glycolytic overactivity may be responsible for hyperinsulinemia early in the disease and can be reduced to restore normal stimulus-secretion coupling. We have previously reported that reducing glucokinase activity in prediabetic mouse islets can restore pulsatility and enhance insulin secretion. Building on this counterintuitive finding, we review the importance of pulsatile insulin secretion and highlight how normalizing glucose sensing in the beta cell during prediabetic hyperinsulinemia may restore pulsatility and improve glucose homeostasis.

## Introduction

An important part of normal insulin secretion is proper glucose sensing; as blood glucose rises, insulin secretion needs to increase in a dose-dependent manner. Insulin secretion also needs to become pulsatile in the proper range of glucose, having small amplitude or non-existent pulses when fasting and larger amplitude pulses postprandially (1, 2). Pulsatile secretion of insulin is a hallmark feature of healthy beta-cells and is caused by oscillations in beta-cell metabolic and electrical activity (3). Pulsatility inhibits glucose production at the liver and maintains high sensitivity of its receptors more effectively than continuous insulin delivery, highlighting the importance of pulsatility in lowering blood glucose levels (1, 4, 5). In beta-cells, oscillations are important for proper insulin granule trafficking and endoplasmic reticulum function, among other proposed benefits (6, 7).

While it is still up for debate if hyperinsulinemia or insulin resistance happens first in the pathogenesis of type 2 diabetes (T2D) (8–12), it is well-established that islets become hypersecretory before glycemic control is lost (13–16). As beta-cell activity increases, glucose sensing becomes aberrant and causes hyperinsulinemia along with the loss of pulsatile insulin secretion within its normal range of glucose stimulation (17–20). The gradual loss of pulsatile insulin secretion precedes the onset of T2D, with type 2 diabetics having no discernable pulses in insulin (21). For this reason, loss of pulsatility has been proposed as a cause of diabetes pathogenesis rather than an effect (22, 23). We have previously shown that endogenous pulsatility is not fully lost in newly diabetic mouse islets, however, their glucose sensing range is left-shifted (18). This left shift refers to islets' increased sensitivity to low levels of glucose, causing them to be pulsatile in hypoglycemic levels of glucose and to be overactive and non-oscillatory in postprandial levels. These factors along with the cumulative effect of worsening hyperinsulinemia and insulin resistance can lead to exhaustion and failure of the cells (17). As beta-cell mass and function decrease, circulating insulin levels decrease and hyperglycemia ensues.

Hyperinsulinemia has been shown as a strong predictor of diabetes in numerous populations (14, 24–26). Current treatment strategies do not diagnose a problem until a patient is prediabetic, at which time islets have already lost an estimated 50–80% of endogenous function (17, 27, 28). Since current diabetes management aimed at lowering A1C has not been successful at reducing the prevalence of T2D, intervening earlier in the progression of the disease may prevent the islets from becoming exhausted and failing in the diabetes-prone population (27, 28). Additionally, hypersecretion of insulin can cause weight gain and make weight loss more difficult since the body is constantly signaling a “fed” state, so lipolysis is decreased and lipid storage is increased (29, 30). Many studies have shown hyperinsulinemia as a driver of insulin resistance (8, 13, 14, 24), in which case attacking the root cause of diabetes and insulin resistance would mean reducing hyperinsulinemia. Targeting early pathological changes in the beta-cell has the potential to prevent or reverse T2D.

## Reducing Glycolytic Activity To Normalize Cellular Function in Overactive Beta Cells

In an effort restore normal function to overactive beta cells, the glucokinase enzyme (GK) is a target with many implications. Calcium-dependent release of insulin granules is largely dependent on the change in the ATP-to-ADP ratio in beta cells (31). Since glucokinase is the first and rate-limiting step of glycolysis, this hexokinase isoenzyme has proven to be an important factor regulating the rate of insulin secretion. Because facilitated glucose transport allows more glucose into the cells than GK can phosphorylate, the enzyme is the true “glucose sensor” that provides the sigmoidal insulin response to physiological levels of blood glucose (32–34).

Due to its ability to control insulin secretion, the sustained catalytic activation of GK in the beta cell has been proposed as a culprit for the increased sensitivity to glucose seen early in the disease (35, 36). Indeed, hyperinsulinemia caused by increased GK activity has been confirmed in several rodent models (35–39). In disease models with increased GK activity, GK mRNA expression can remain unchanged but the enzyme will have an augmented response to glucose, and thus a lower threshold for insulin secretion (35, 38, 40). Other studies have reported an increase in GK at the protein level because posttranslational modifications can stabilize the enzyme to slow turnover and reduce oxidative inactivation (41–44). Another set of studies showed that prolactin increases GK expression and insulin secretion which may partially explain the increased beta-cell activity seen in pregnancy (45, 46). Enzymatic activity can be enhanced post-translationally by numerous factors including increased cytoplasmic calcium, increased plasma free fatty acids, insulin signaling, and the cooperative binding of glucose (37, 47–51); all of which become elevated early in the disease. Therefore, GK may play an etiological role driving beta-cell exhaustion in susceptible populations.

We propose that peripheral influences cause GK to be more sensitive to glucose, as opposed to underlying mutations in GK driving islet overactivity and exhaustion. In this state of heightened glucose sensitivity, islets secrete insulin in sub-stimulatory glucose but cannot secrete enough insulin in postprandial glucose levels (52, 53). Fasting hyperinsulinemia can be detected 10–20 years before T2D diagnosis (54), indicating that a slow but progressive increase in GK activity would be a prime suspect. A potential trigger for a left shift in glucose sensitivity is the prolonged exposure to excess glucose and lipids seen in individuals at risk for T2D. Studies show that exposing islets to high glucose over a period of days can potentiate insulin secretion in response to glucose (53, 55, 56), and certain durations and types of free fatty acids can do the same (57–59).

Treating islets in high glucose increases GK V_max_, a change that is sustained when normal glucose levels are restored (41, 43, 60). Since GK expression remains relatively constant, the shift in GK activity is thought to be caused by posttranslational modifications (43). While the S_0.5_ is unaltered, raising the V_max_ augments the insulin response to glucose and causes a left shift in the EC_50_ of insulin secretion (41). This is contrary to small molecule GK activators that decrease the S_0.5_ and may or may not increase the V_max_ (61, 62). A constant S_0.5_ with an increased V_max_ causes a steeper slope and inflection point where glycolytic flux and insulin secretion would be amplified. Numerous posttranslational modifications have been found to increase the activity of GK including interaction with PFK-2/FBPase-2, S-nitrosylation, interaction with BCL 2-associated death promoter (BAD), GK ubiquitination, and SUMOylation, as reviewed in (63).

While these modifications act on the order of minutes to allow for cooperative kinetics, some modifications may be sustained and augment glucose phosphorylation. Collectively, these modifications can cause more GK to be available in the cytosol in a highly active conformation, independently of the amount of glucose present in cell. For example, S-nitrosylation dissociates GK from insulin granules, allowing for more GK in the cytosol and increased cellular GK activity (51, 64). Increasing GK expression left shifts the response curve, so having more available GK in the cytosol may do the same (65). S-nitrosylation of GK can be enhanced by GLP-1 which partially mediates its insulinotropic effects and lowers the set point for insulin secretion (68). As another example, PFK-2/FBPase-2 interaction with GK increases its V_max_, indicating that increased expression or protein binding activity of this bifunctional enzyme could potentially explain the increased V_max_ and unaltered S_0.5_ after treating islets in high glucose (41, 43, 66, 67), although a causative role of PFK-2/FBPase-2 has not been established. Numerous posttranslational mechanisms can shift GK to a more active state which may be brought on by excess nutrient load (57, 58), the need to adapt to insulin resistance and hyperglycemia (55, 60), or another mechanism not yet identified (12). In sum, GK activity can be increased by posttranslational modifications that increase the amount of the enzyme in the cytoplasm or change its conformation into a more active state. Such modifications can cause an increase in V_max_, which is consistent with changes found after treating islets in high glucose.

**Figure 1:**
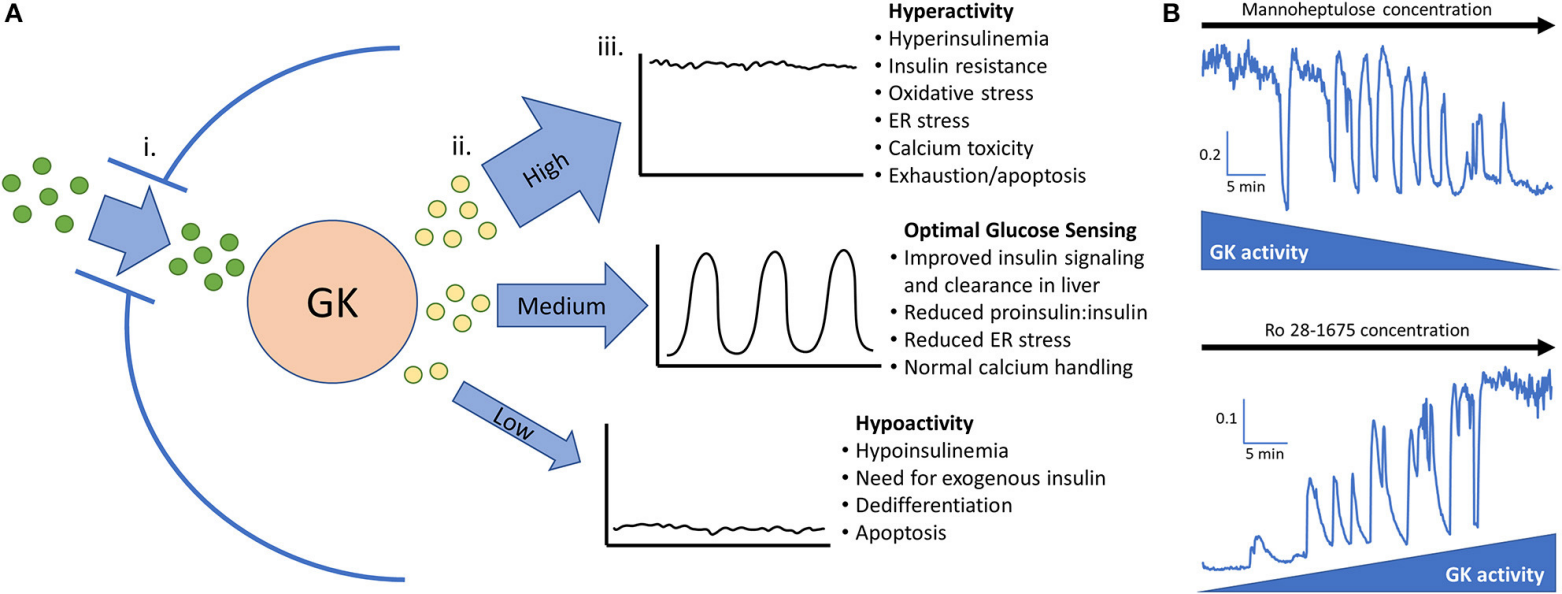
**(A)** Glucokinase phosphorylates glucose and feeds the endogenous oscillator. (i). Permissive levels of glucose enter the beta cell through facilitated glucose transport from the blood. (ii). Glucokinase phosphorylates varying amounts of glucose depending on the quantity and activity level of the enzyme along with the blood glucose level. Glucokinase activity levels are largely dependent on the amount of glucose present in the cell but can be augmented in pathological conditions such as hyperinsulinemia and diminished late in the disease process. (iii). High, medium, or low levels of phosphorylated glucose move to the next steps of glycolysis and can activate the endogenous oscillator in the correct range (~5–20 mM glucose in islets from non-diabetic mice). **(B)** *In vitro* evidence that GK has high control over islet activity and oscillations. Calcium imaging of islets isolated from CD-1 control mice give proof of concept that reducing glucokinase activity in 20 mM glucose can restore pulsatility. Conversely, stimulating glucokinase activity in 3 mM glucose can generate pulsatility and increase intracellular calcium to the point that no calcium oscillations can occur. This adds support that glucose transporters allow permissive levels of glucose into the cell for glucokinase to be the true glucose sensor. Scale bars represent changes in intracellular calcium (fura-2 am 340/380 nm) as a function of time (min). This figure is based on previously published data in (19).

The GK enzyme in the beta-cell completes the first step of glycolysis by phosphorylating glucose. After this rate limiting step is completed, phosphorylated glucose is fed to phosphofructokinase (PFK) which is believed to be the key enzyme responsible for slow glycolytic oscillations (3, 69). Oscillations can occur only when GK activity levels are in a specific range; if the glucose phosphorylating capacity is above or below this range, pulsatile insulin secretion is diminished (Figure 1A) (19, 69). This range is estimated to be ~5–20 mM glucose in islets from non-diabetic mice (70, 71). Shifting GK activity by altering cellular content or enzyme conformation will also shift the threshold of insulin secretion and subsequently the range at which oscillations can occur (55, 58). Pharmacologically decreasing GK activity in high glucose can restore oscillations, while increasing GK activity in low glucose can also stimulate oscillations (Figure 1B). This finding was originally reported in (19).

Previous pharmacologic interventions have aimed to activate glucokinase activity to stimulate insulin secretion (72). This concept is based on studies that show a profound decrease in GK and GLUT1-3 expression in the later stages of the disease, likely due to dedifferentiation caused by long term abuse of the islets (17, 73–75). Glucokinase activators have not been successful in long term clinical trials due to lack of durability (72, 76). A similar situation to the pharmacological activation of GK is seen in some cases of persistent hyperinsulinemic hypoglycemia of infancy where a mutation in GK lowers its S_0.5_ for glucose, which can lead to beta-cell dysfunction and diabetes later in life (77). Some case studies have shown a link between GK activating mutations and early onset diabetes, providing evidence that long-term beta-cell overactivity caused by augmented GK activity may contribute to beta-cell dysfunction (78–80). Alternatively, since GK catalyzes the rate-limiting step of glycolysis, reducing its activity will decrease downstream glucose-stimulated insulin secretion. Intervening at this vital point can feed PFK the correct amount of glucose-6-phosphate to produce oscillations.

A competitive inhibitor of GK is D-mannoheptulose (MH), a naturally occurring sugar found in the avocado whose implications in diabetes have been studied for decades (81–83). Due to its inhibitory nature, most studies have used MH to counter hyperinsulinemic hypoglycemia caused by diseases such as insulinoma, though more recent studies suggest it may have other uses (18, 84, 85). We recently reported that MH can restore normal glucose sensing in pancreatic islets from newly diabetic mice. Decreasing glycolysis ~20–40% by treating islets acutely or overnight with MH can normalize left-shifted glucose sensitivity (18, 19). By decreasing cellular activity only slightly, pulsatility can be restored within the normal range of blood glucose. Along with these findings, we discovered that insulin secretion, ATP levels, and NAD(P)H flux were paradoxically increased in diabetic islets treated with MH (19). This finding is counterintuitive considering GK activators have been developed to increase insulin secretion but a GK inhibitor at the appropriate concentration actually enhanced insulin secretion. A paradoxical increase in insulin secretion has also been shown when reducing the glucose level perfused through pancreases from diabetic rats (39) and when treating obese mice with MH *in vivo* (81). Paired *in vitro* data and mathematical modeling show that treating islets in high glucose causes a sustained increase in GK V_max_ and that adding MH can partially correct the curve by reducing the V_max_ and increasing the S_0.5_ (43, 86). While these studies give proof of concept that normal stimulus-secretion coupling can be restored by reducing GK activity, MH likely holds little value clinically since it is active in the millimolar range and would also inhibit hepatic glucokinase. Nonetheless, MH was able to normalize glucose sensitivity, restore oscillations, and increase insulin secretion in islets from newly diabetic mice.

Genetic mutations have given us examples of what happens when GK activity is chronically higher or lower than it should be. Inactivating mutations in the GK gene cause maturity onset diabetes of the young type 2 and permanent neonatal diabetes mellitus, whereas activating mutations cause some forms of congenital hyperinsulinism (87, 88). These mutations can alter the S_0.5_ and K_cat_, which effectively shifts the threshold for insulin secretion and results in various degrees of hyper or hypoglycemia (89, 90). Some mutations may restrict posttranslational modifications such as the interaction with PFK-2/FBPase-2 or S-nitrosylation (63). Whereas glucokinase diseases are present from birth, our approach would be moving the high glycolytic activity seen in hyperinsulinemia back down to a typical range, thereby normalizing stimulus-secretion coupling in the beta cell. However, this remains hypothetical and may be difficult to titer using inhibitory compounds *in vivo*.

## Benefits of Normalizing Islet Function To Restore Pulsatility

**Figure 2:**
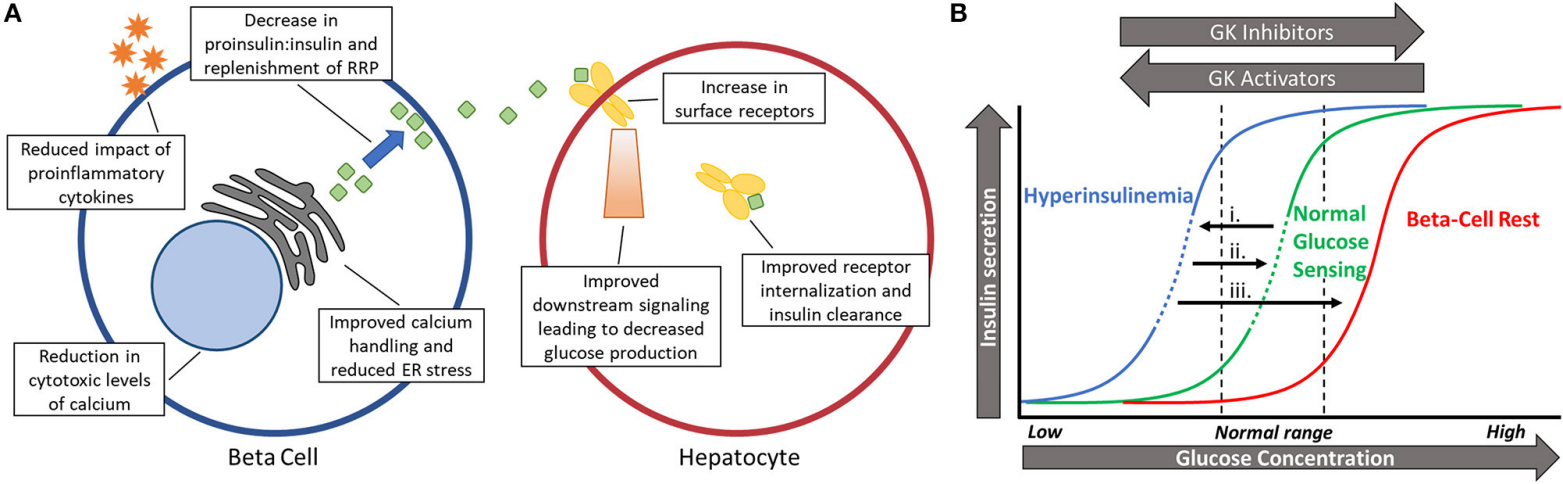
**(A)** Schematic showing the benefits of restoring pulsatility in the beta-cell and hepatocyte. Numerous beneficial effects may take place in the beta cell (left) and hepatocyte (right) after glucokinase activity is normalized and pulsatility is restored. **(B)** Shifting glucose dose-response curves based on changing levels of glycolytic activity. (i). Long before diabetes is diagnosed, glucose sensing becomes left-shifted due to excess glucose phosphorylation by glucokinase. This leads to hyperinsulinemia and lowers the threshold for oscillations (dashed portion of line). (ii). Slightly reducing beta-cell activity (right-shift) by reducing glucokinase activity can restore normal glucose sensing and pulsatility in an appropriate range of glucose (dashed portion of line). (iii). Full inhibition during beta-cell rest does not permit oscillations and almost completely stops insulin secretion in physiological blood glucose levels.

Normalizing glucose sensing in diabetic islets may provide numerous benefits at the cellular level (Figure 2A), with improved intracellular calcium handling being particularly valuable. Islets from prediabetic mice are overstimulated and have chronically elevated basal intracellular calcium levels in fasting levels of glucose (18, 91), which can lead to cytotoxicity and subsequent apoptosis through calcium dependent proteases (92–94). Other genes shown to be affected by either high calcium or membrane depolarization include those associated with dedifferentiation, loss of cell adhesion, and beta-cell failure (95, 96). By reducing glycolysis and the downstream influx of calcium, gene expression associated with viability may be maintained. Additionally, cellular stress caused by proinflammatory cytokines can be reduced once glucose sensing is corrected since hypersensitivity to glucose magnifies these negative effects (18, 94, 97).

Along with right-shifting glucose sensing comes a return of oscillations and pulsatile insulin secretion within postprandial glucose levels. In a study of islets isolated from db/db mice in the early stages of diabetes, the increased insulin secretion observed after treatment with MH correlated closely with the amplitude of intracellular calcium oscillations (19). It remains to be seen if this is due to a decrease in the ratio of proinsulin-to-insulin secretion caused by a reduction in ER stress or by allowing more time for replenishment of the readily releasable pool between pulses (6, 7, 98, 99). Regardless, pulsatility has clear implications in proper insulin secretion in rodents and humans alike (20, 22, 100–102). Another beneficial facet of insulin pulsatility is the proper suppression of glucagon secretion from alpha cells through paracrine signaling (103, 104). Decreased insulin pulse mass early in the disease causes impaired intra-islet communication and postprandial hyperglucagonemia (103–105). Insufficient amounts of insulin paired with excess glucagon is a recipe for hyperglycemia.

Outside of the islets, pulsatile insulin secretion at the liver is paramount for a proper physiological response (Figure 2A). Insulin delivered in pulses has been repeatedly shown to block the production of glucose better than continuous delivery (5, 106). Since the liver is a central component of blood glucose regulation, more efficiently blocking glucose production can have beneficial effects on the whole body. In fact, restoring pulsatility instead of allowing constantly high levels of insulin secretion has the potential to upregulate insulin receptors at the liver, thus reducing systemic insulin resistance by improving hepatic insulin clearance (107, 108). Therefore, pulsatility has the potential to physiologically increase insulin sensitivity in the liver and peripheral tissues instead of pharmacologically, such as with metformin. Sufficient transcriptional expression of GK in the liver is also dependent on pulsatile insulin signaling, showing decreased expression when pulsatility is lost (5). While glucose is the primary regulator of pancreatic GK expression, insulin signaling has the greatest stimulatory effect on GK in the liver, creating a cross-talk between glucokinase regulation in separate organs (33, 40). GK in the liver acts as a key regulator of net glucose flux so maintaining high expression of the enzyme can increase glucose storage and decrease glucose production (109, 110).

During treatment with inhibitory compounds, glucose sensitivity is right shifted and pulsatile instead of hyperactive, which may simultaneously lower secretory stress on the cell while improving whole-body glycemia. It remains to be seen if this can be done *in vivo* to compare directly to other methods of beta-cell rest. If successful, restoration of pulsatility could provide better signaling at the liver, less proinsulin secretion due to improved insulin granule trafficking, and decreased apoptosis as a result of normalized intracellular calcium levels. At least one clinical study has successfully shown that reducing islet activity can restore pulsatile insulin secretion, indicating that the ability is not lost in newly diabetic patients (111). It has also been shown that hyperinsulinemia precedes insulin resistance in the development of T2D (13, 24, 112). Therefore, if hyperinsulinemia and islet overactivity is corrected early in the disease, insulin resistance may not progress as rapidly. In this way, modifying cellular metabolism of the beta cell may improve whole-body metabolism.

## Clinical Implications

A considerable amount of research has examined the beneficial effects of resting beta cells. The theory of beta-cell rest has been studied since the 1940's when patients with type 1 diabetes showed brief remission after a course of intensive insulin therapy (113, 114). Now known as the “honeymoon phase,” this return of endogenous insulin secretion gave initial insight into the cellular benefits of reducing secretory demand. In the 1970's diazoxide was used to directly rest beta-cells in patients with T2D which led to a significant increase in insulin secretion following treatment (115). Since then, the importance of maintaining beta-cell function in T2D has become increasingly evident, and several new mechanisms of resting the cells have emerged (28, 116, 117).

In clinical studies, beta-cell rest involves decreasing secretory stress on the beta-cells, hoping for a return of function that continues once the treatment is stopped. The period of rest is thought to allow time for replenishment of the readily releasable pool of insulin (99, 111), a reduction in oxidative stress (118–120), or recovery of normal GK activity levels (41, 43, 121). Many clinical studies discussing beta-cell rest focus on the use of oral medications that restore euglycemia, therefore indirectly lowering the demand on the beta cell to secrete insulin while also decreasing the effects of glucotoxicity. While lowering blood glucose is beneficial to peripheral tissues, classes of medications such as second-generation sulfonylureas and incretin mimetics achieve blood glucose control mainly by increasing beta-cell activity, and therefore should not be regarded as agents of beta-cell rest. Sulfonylureas have been shown to increase the rate of beta-cell functional decline along with inducing apoptosis, highlighting the importance of resting instead of stimulating beta cells (122, 123).

The ability of medications to induce beta-cell rest have been recently reviewed elsewhere and highlight the many benefits of decreasing secretory stress (116, 122, 124). Collectively, these studies have shown a modest increase in beta-cell function during and after treatment, which is typically lost as the disease progresses and the use of additional medications and insulin becomes necessary. The short-lived benefits after providing transient beta-cell rest may be enhanced if the objective is to restore normal function to the islets long-term rather than fully inhibit them. Additionally, in most studies regarding beta-cell rest the intervention begins after patients have been diagnosed with T2D. If implemented early enough, restoring normal function could prevent diabetes before it progresses to the point of apoptosis and dedifferentiation causing loss of beta-cell mass and downregulation of critical beta-cell genes (122, 125). Otherwise, susceptible patients will have a lifetime dependency on insulin. Perhaps the early use of an SGLT2 inhibitor or lifestyle intervention can reduce hyperinsulinemia by reducing blood glucose with no stimulatory effect on the beta cell. Further investigation into the beneficial aspects of beta-cell rest and how to best implement it is warranted.

## Discussion and Future Research Avenues

The beta cell has repeatedly shown its ability to regain function when rested or removed from a toxic environment (126, 127). Perhaps restoring healthy levels of function by normalizing glucokinase activity can aid in proper insulin secretion without overworking the cells. GK is a unique enzyme due to its high control over insulin secretion. It has implications early in diabetes acting to left-shift islet's sensitivity to glucose, and late in the disease to contribute to glucose insensitivity once dedifferentiation occurs. GK also acts as an important regulator of glycolytic flux to allow PFK and other downstream mechanisms to produce oscillations. Although MH has shown proof of concept *in vitro* that oscillations can be restored, the competitive inhibitor is effective in the millimolar range which limits its clinical potential. Additionally, MH would inhibit GK present in the liver which would cause impaired glucose storage and lead to a rise in blood glucose (83). However, this basic research finding opens an avenue of research into other methods of decreasing GK activity during hyperinsulinemic states since most pharmacological research thus far has focused on activating the GK enzyme late in the disease. Other compounds that reduce GK activity more selectively and potently in overactive beta cells would hold value. Given the importance of pulsatility in insulin signaling and degradation at the liver, targeting the loss of pulsatility could delay or avoid a key pathological change in the progression of diabetes.

Alternatively, other cellular components of glucose-stimulated insulin secretion can be targeted to directly rest the islets. Some studies have shown K_ATP_ channel conductance to be decreased as part of the left-shift phenomenon, so K_ATP_ channel activators may be another useful target that act independently of glucose (55, 56). In addition, the long-acting somatostatin analog octreotide can effectively reduce hyperinsulinemia, which also allowed for enhanced weight loss in patients (128). Another clinical study showed that pulsatility could be restored temporarily after overnight inhibition of insulin secretion with somatostatin (111); although we propose a long-term reduction in overactivity rather than short-term full inhibition. Whatever the mechanism, lowering activity through an agent acting directly on the beta-cell would be of great benefit and has been proposed elsewhere (24, 29, 129). Future studies in our lab will look at the best methods of restoring normal glucose sensing and move to study the method *in vivo*.

An obvious caveat to our therapeutic concept is that decreasing insulin secretion by directly inhibiting the beta-cell in an insulin resistant individual would cause blood glucose to increase rapidly. However, in our studies we have seen a paradoxical increase in insulin secretion when GK activity was slightly decreased due to a strong correlation to pulse amplitude (19). Similar patterns of insulin release were reported as glucose was lowered in the perfused pancreas of Zucker Diabetic Fatty rats (39). Additionally, if pulsatility is restored in a normal range of glucose sensing, the liver would be able to transduce insulin signaling more effectively and clear more insulin in the first pass before the rest is released into circulation. Other studies that propose hyperinsulinemia as the primary driver of insulin resistance allude to the chance of decreasing hyperinsulinemia as a mechanism to decrease insulin resistance (8, 29, 30). Decreasing insulin secretion an appropriate amount with diazoxide can reduce insulin resistance and improve weight loss without the loss of glycemic control (13, 29, 30). This shows that hyperinsulinemia is not necessary to maintain euglycemia, but rather this level of insulin is excessive (8).

Another option is to treat the two main components of T2D pathogenesis separately. Beta-cell oversecretion can be managed with an inhibitor while the potential increase in blood glucose is managed with an oral hypoglycemic agent until metabolic homeostasis is restored. This would cause a beneficial right-shift back to normal glucose sensing while maintaining blood glucose and insulin levels in a normal range. Again, this two-pronged approach may not be necessary if the liver responds appropriately (129), and the caveats to our proposals *in vivo* can be managed with existing therapies as needed. An additional consideration is that T2D does not only involve a functional change in GK activity. It is a multifaceted disease with many peripheral issues that occur simultaneously to those seen in the beta-cells. Pathogenic changes outside the beta-cell may proceed to the point that insulin secretion is still insufficient to maintain glucose homeostasis.

The current methods of treating T2D have not been successful in reducing the prevalence of disease so novel avenues need to be explored. Current drug classes and treatment strategies aim to reduce HbA1C while suggesting healthy lifestyle habits. While this avoids the complications associated with high blood sugar it most often fails and necessitates multiple drug combinations or exogenous insulin (130). Thus, we have proposed two main points. First, treatment targeting pathological changes in beta-cells should begin during the hyperinsulinemic stage before beta-cell mass and function are irreversibly compromised. Second, GK provides an attractive target for normalizing function and restoring pulsatility early in the disease, and that it is at least partially responsible for increased islet sensitivity to glucose and hyperinsulinemia (Figure 2B). Through sharing this perspective on diabetes treatment, we hope to open new avenues of basic and translational research to lessen the incidence of type 2 diabetes and metabolic syndrome.

## Author Contributions

CN initiated the writing of the manuscript. NW wrote a large majority of the manuscript with continual review from CN. Both authors approved the final edition.

## Conflict of Interest

The authors declare that the research was conducted in the absence of any commercial or financial relationships that could be construed as a potential conflict of interest.
